# Population Analysis of Adverse Events in Different Age Groups Using Big Clinical Trials Data

**DOI:** 10.2196/medinform.6437

**Published:** 2016-10-17

**Authors:** Jake Luo, Christina Eldredge, Chi C Cho, Ron A Cisler

**Affiliations:** ^1^ Center for Biomedical Data and Language Processing College of Health Sciences, Department of Health Informatics and Administration University of Wisconsin-Milwaukee Milwaukee, WI United States; ^2^ Department of Health Informatics and Administration College of Health Sciences University of Wisconsin-Milwaukee Milwaukee, WI United States; ^3^ College of Health Science University of Wisconsin-Milwaukee Milwaukee, WI United States; ^4^ Department of Family and Community Medicine Medical College of Wisconsin Milwaukee, WI United States; ^5^ Center for Aging and Translational Research University of Wisconsin-Milwaukee Milwaukee, WI United States; ^6^ Center for Urban Population Health Milwaukee, WI United States; ^7^ School of Medicine and Public Health University of Wisconsin-Madison Milwaukee, WI United States; ^8^ Zilber School of Public Health University Wisconsin-Milwaukee Milwaukee, WI United States

**Keywords:** big data analysis, adverse events, clinical trial data, population health, clinical trial safety, data processing and integration

## Abstract

**Background:**

Understanding adverse event patterns in clinical studies across populations is important for patient safety and protection in clinical trials as well as for developing appropriate drug therapies, procedures, and treatment plans.

**Objectives:**

The objective of our study was to conduct a data-driven population-based analysis to estimate the incidence, diversity, and association patterns of adverse events by age of the clinical trials patients and participants.

**Methods:**

Two aspects of adverse event patterns were measured: (1) the adverse event incidence rate in each of the patient age groups and (2) the diversity of adverse events defined as distinct types of adverse events categorized by organ system. Statistical analysis was done on the summarized clinical trial data. The incident rate and diversity level in each of the age groups were compared with the lowest group (reference group) using *t* tests. Cohort data was obtained from ClinicalTrials.gov, and 186,339 clinical studies were analyzed; data were extracted from the 17,853 clinical trials that reported clinical outcomes. The total number of clinical trial participants was 6,808,619, and total number of participants affected by adverse events in these trials was 1,840,432. The trial participants were divided into eight different age groups to support cross-age group comparison.

**Results:**

In general, children and older patients are more susceptible to adverse events in clinical trial studies. Using the lowest incidence age group as the reference group (20-29 years), the incidence rate of the 0-9 years-old group was 31.41%, approximately 1.51 times higher (*P*=.04) than the young adult group (20-29 years) at 20.76%. The second-highest group is the 50-59 years-old group with an incidence rate of 30.09%, significantly higher (*P*<.001) when compared with the lowest incidence in the 20-29 years-old group. The adverse event diversity also increased with increase in patient age. Clinical studies that recruited older patients (older than 40 years) were more likely to observe a diverse range of adverse events (*P*<.001). Adverse event diversity increased at an average rate of 77% for each age group (older than 30 years) until reaching the 60-69 years-old group, which had a diversity level of 54.7 different types of adverse events per trial arm. The 70-100 years-old group showed the highest diversity level of 55.5 events per trial arm, which is approximately 3.44 times more than the 20-29 years-old group (*P*<.001). We also observe that adverse events display strong age-related patterns among different categories.

**Conclusion:**

The results show that there is a significant adverse event variance at the population level between different age groups in clinical trials. The data suggest that age-associated adverse events should be considered in planning, monitoring, and regulating clinical trials.

## Introduction

Clinical trials explore and evaluate the safety and effectiveness of clinical interventions. Many clinical trial interventions are experimental, and thus they have greater risks to adversely affect the health of the participants in comparison to standard clinical practice. The adverse events data in this study were extracted from ClinicalTrials.gov. An adverse event is defined by ClincialTrials.gov as unfavorable changes in health during clinical trials, including abnormal laboratory findings [1]. Serious adverse events include events that result in death, disability, birth defects, inpatient hospitalizations, prolongation of hospitalization, or life-threatening conditions.

Adverse event reporting is a critical measurement for estimating the safety of new treatments and new drug therapies. According to the literature, adverse events could be one of the leading causes of death in the United States [2]. Serious adverse events could lead to hospitalization, life-threatening symptoms, or even patient death [3,4]. Studies also found that adverse reactions are a significant cause of injury in children [4]. Therefore, analyzing the pattern of adverse events in clinical studies has a great importance to public health and significant value to clinical research.

Drug studies have shown the importance of age as a factor influencing the incidence of adverse events in clinical studies. For example, among heart failure patients, the adverse event incidence of digoxin increases progressively with age, from 1.7% among patients younger than 50 years old to 5.4% among patients aged older than 80 years. Hospitalizations from digoxin toxicity also increase with age [5]. Additionally, a recent study found that age and obesity are significant risk factors for adverse events after hip arthroplasty treatment [6]. Furthermore, a clinical trial involving inhaled corticosteroids for treating children with asthma found that cough and perioral dermatitis are more frequent in children younger than 6 years old, while hoarseness is more frequent in older children [7]. These studies analyzed the association between individual treatments and adverse events. However, currently there is a lack of population health level analysis of adverse event association with patient age.

The objective of this study was to compare the incidence rate and diversity of adverse events in clinical trials among different age groups, revealing potential adverse event disparities between the patient age groups. In comparison to standard adverse event analyses in individual clinical trials, this study focused on comparing adverse events between different age groups across 17,853 trials and 6,808,619 participants that could have different interventions. The adverse events observed during clinical trials are not necessarily induced by the trial intervention. The adverse events could be inherited from the recruited participant population, which is a crucial factor to consider for planning and conducting clinical trials. We aimed to reveal adverse event risks and patterns on the population level across different age groups in clinical trials to inform investigators for use in future clinical trial preparation. Currently, there is a gap in this level of knowledge.

## Methods

### Data Extraction and Preparation

The source data in this study were extracted from ClinicalTrials.gov, which is the largest public clinical trial repository [1,8]. We downloaded 186,339 clinical trial studies submitted from 2000 to 2014, from which we extracted 17,853 studies that published the actual outcome results. Using an XML parser [9] developed to extract data elements from clinical trial reports, we collected the clinical trial title, sponsor type, intervention, participant age, arm group, and adverse events. In this study, we focused on analyzing the age categories and their association with adverse events.

We collected a total of 6,808,619 clinical trial participants and approximately 11,000 types of adverse events. Based on the reported mean age, the study population in each of the trial arms was categorized into eight age groups in 10-year increments except for the 70 to 100 years-old group. The adverse events were originally encoded in different terminologies in the reports, such as the Medical Dictionary for Regulatory Activities (MedDRA) [10,11], the World Health Organization Adverse Reactions Terminology [12], and the *International Classification of Diseases, Ninth Revision* [13]. We mapped the extracted reported adverse events into Unified Medical Language System (UMLS)-based standardized concepts using the MetaMap application [14,15] to normalize the terminology across different trials. All collected adverse events were classified into the 26-group MedDRA system organ classes (SOCs) [10] based on the classification provided by ClinicalTrials.gov. The extracted data were stored in the Clinical Trial Adverse Event Database for analysis [9]. We analyzed the association between age and adverse event from two perspectives: the incidence rate of adverse events and the diversity of adverse events. Statistical analysis was done on the summarized data using the Excel 2016 (Microsoft Corp) statistical package.

### Analysis of Adverse Event Incidence

The incidence of adverse events is commonly used to evaluate the safety of a new treatment. If an adverse event has a high incidence rate in clinical trials, this indicates the event is more likely to occur among the study patients. For each of the adverse events in each trial arm, we collected the total number of affected patients and the number of at-risk patients. The incidence per study is calculated as the percentage of at-risk patients affected by adverse events. Many population-level studies have analyzed the adverse event incidence rate among different age groups and clinical settings such as in-hospital, outpatient, after discharge, and nursing homes. However, in the past, research on adverse events in clinical trial studies has primarily focused on individual drug and selected participant groups. Population-level analysis of adverse events across different age groups and interventions in clinical trials is lacking. The objective of this study is to fill this knowledge gap by providing systematic analysis of adverse event incidence in clinical trials by comparing the incidence and diversity of adverse events in different age groups across clinical trials.

### Analysis of Adverse Event Diversity

Adverse event diversity examines how many distinct types of adverse events (eg, cardiac failure, depression, patient death) occur in clinical studies. The diversity of adverse event occurrence is an important factor for estimating intervention risks; however, it is often overlooked. When a study therapy is associated with a high diversity of adverse events in a population group, the complexity and cost of developing effective procedures to prevent and treat the adverse events could also increase [16,17]. To compare the adverse event diversity, we categorized the participant population in each of the trial arms according to the age groups. Then, we summarized the distinct types of adverse events that occurred in each of the age groups in the trial. The adverse event diversity was calculated on the trial arm level. For example, if a trial arm for a study has the adverse events heart failure, dizziness, and nausea, then the diversity of this trial arm would be 3. The mean of the adverse event diversity in each of the age groups was calculated as the number of distinct adverse event types divided by the number of trial arms of the age group, which indicates the average number of distinct adverse events in each of the trial arms. To further assess adverse event diversities in different organ systems, the adverse events were categorized into the 26 MedDRA SOCs. We then compared the adverse event diversity in each of the organ classes across the eight age groups.

## Results

### Incidence of Adverse Event

**Figure 1:**
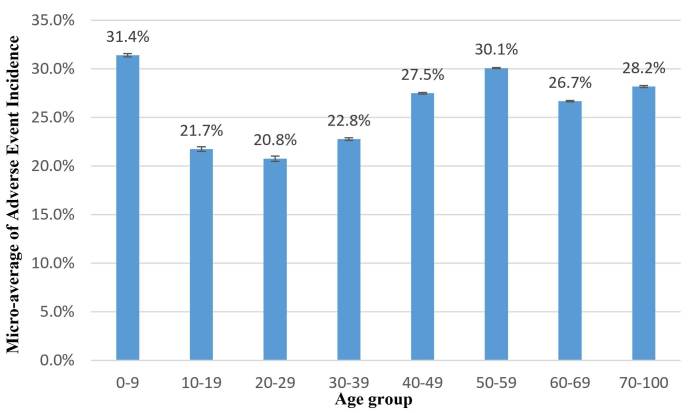
Adverse event incidence rate with different age groups. (X-axis: age group; Y-axis: micro-average of adverse event incidence in an age group; confidence intervals are shown on the bar.).

Figure 1 shows the average adverse event incidence rate of each of the age groups. The total number of affected patients and the corresponding MedDRA SOCs are also shown in Figure 1. The results show that the 20 to 29 years-old group has the lowest adverse event incidence rate of 20.76%. The highest group is the 0 to 9 years-old group, with an incidence rate of 31.41% and *P*=.02 (*P*<.05, *t* test) when compared with the lowest group, 20 to 29 years-old. The risk difference between the 0 to 9 years-old and 20 to 29 years-old groups is 10.6% (SE 0.00070). The results indicate that young children are more susceptible to adverse events than the young adult reference groups on a population level. The second highest group is 50 to 59 years-old, with an incidence rate of 30.09% and *P*<.001 (*t* test) when compared with 20 to 29 years-old group. The risk difference between the 50 to 59 years-old group and the 20 to 29 years-old group is 9.3% (SE 0.00059). Generally, the incidence rate increases with age in the nonpediatric groups (aged 30 years and younger). However, the groups of patients aged older than 60 years see a small drop in adverse event incidence, but the exact reason for this is still not clear. In general, Figure 1 shows a nonlinear trend appearance with peaks at the 0 to 9 years-old and 50 to 59 years-old groups.

**Figure 2:**
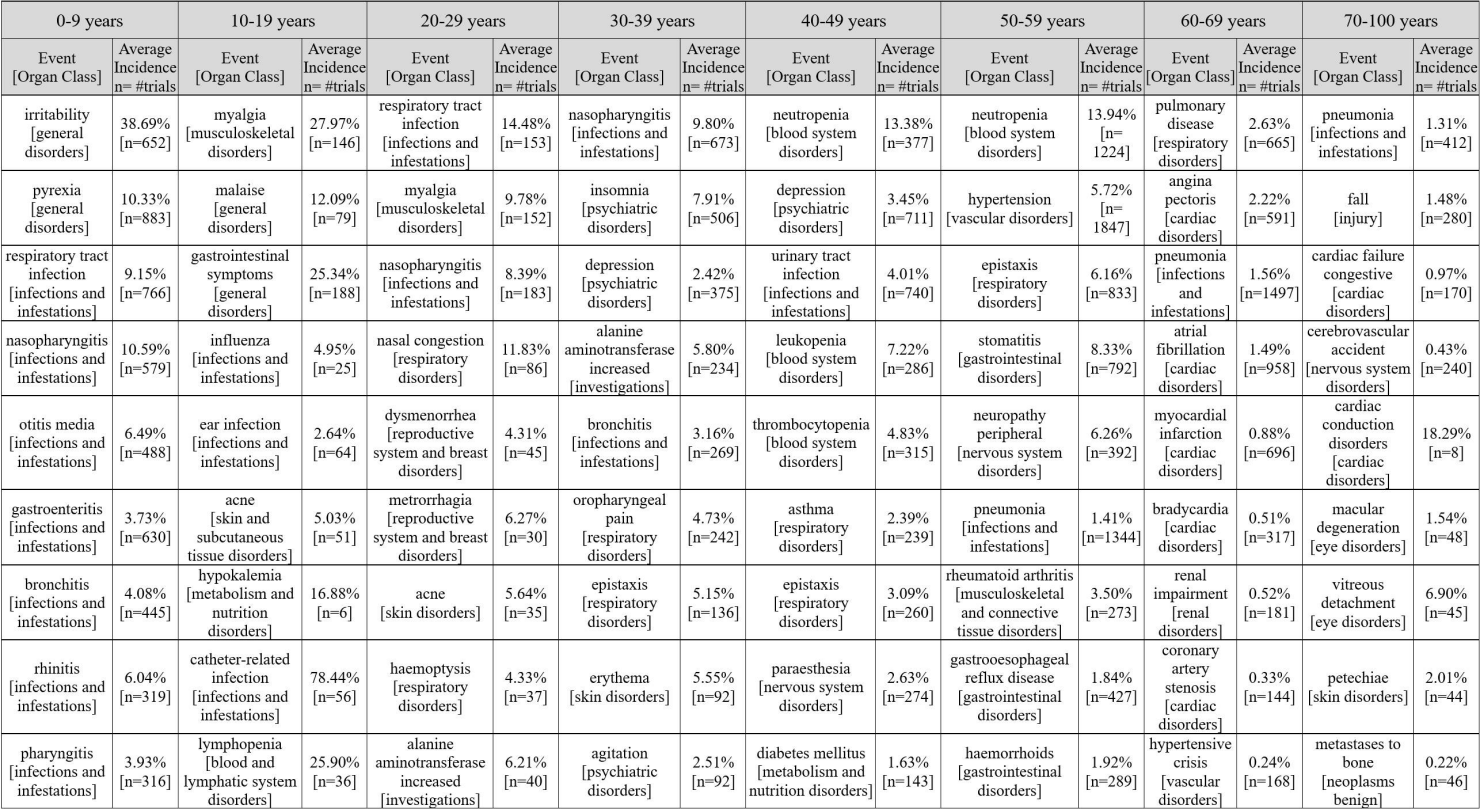
Top significant adverse event examples across clinical trials in each age group (*P*<.01). Shared nonserious events were filtered out.

Figure 2 lists the top adverse event examples in each of the age groups that show higher incidence rate across different clinical trials when compared to other age groups. Within each age group, we selected the top events that show significance (*P*<.01, *t* test) when compared with the comparison group. The comparison group consists of trials that reported the same event but with patients who were not in the same age group. For example, given the 0 to 9 years-old group and adverse event pharyngitis, we can find 316 trials that have an average incidence rate of 3.93% for the age group. The comparison group includes 299 trials that reported the same event among 10 to 100 years-old patient groups (at an average incidence rate of 2.17%) . Using *t* tests to compare both groups, we have *P*<.01, which statistically shows that pharyngitis is significantly higher among the young child group across clinical trials. The results in Figure 2 indicate that individual adverse events can have a significant disparity in term of incidence rate across different age groups. Commonly shared nonserious events are filtered (see Multimedia Appendix 1). The data also indicate there are strong patterns of adverse events in each of the age groups. For example, the 0 to 9 years-old group shows a significant number of adverse events in infection and infestation: 7 out of the top 9 events in the group are infection events. Young adults (20-29 years-old group) show adverse events with the reproductive system and musculoskeletal system; older adults (30-49 years-old group) show a higher level of adverse events in psychiatric and respiratory disorder categories. Blood system events and gastrointestinal events are higher in the 50 to 59 years-old group, and the oldest patients (60-100 years-old group) generally are at significantly higher risk for cardiac and vascular disorders than other age groups.

### Diversity of Adverse Events

**Figure 3:**
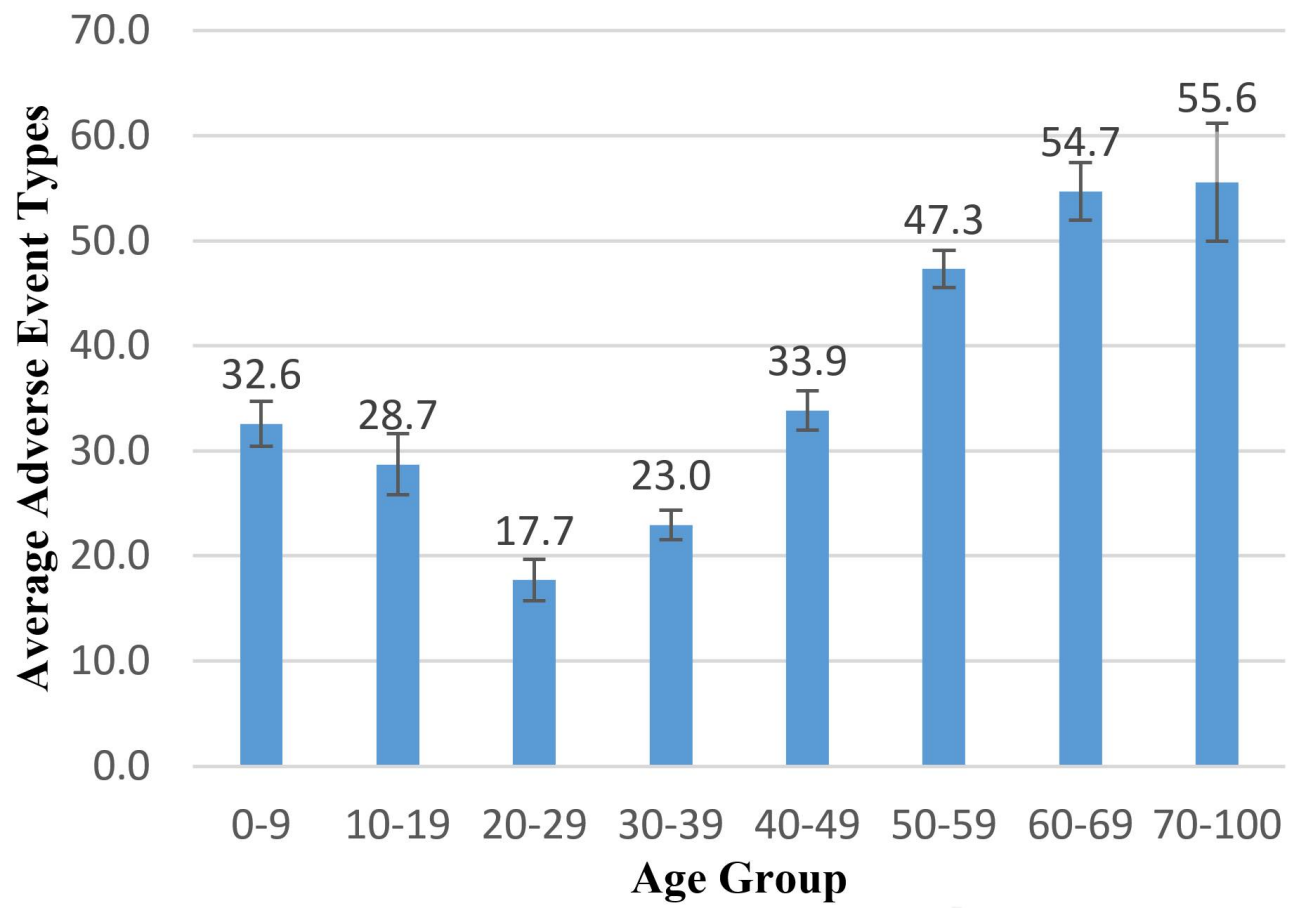
Diversity of adverse events among different age groups. (X-axis: age groups, Y-axis: average adverse event types; confidence intervals are shown on the bar.).

Approximately 11,000 distinct adverse event types were observed in 6,808,619 participants. The adverse event diversity analysis was performed on the trial arm level, in which a group of patients received the same clinical intervention (eg, drug, surgery). We first analyzed the diversity among different groups of patients. Figure 3 shows that older groups of patients (aged 50 years and older) have a much higher diversity level of adverse events compared with the younger groups. The lowest diversity group is the 20 to 29 years-old young adult group. The group of young children (0-9 years-old group) also showed higher adverse event diversity than the young adult (20-29 years-old) group. On average, the young adult group observed 17.71 events/arm (95% CI 15.72-19.70, SE 1.02) of distinct adverse events. The young children group showed 32.58 events/arm (95% CI 31.49-34.71, SE 1.09) of distinct event types on average, which is approximately 1.84 times greater on average than the young adult group (*P*<.001). In comparison to the lowest affected 20 to 29 years-old group, the adverse event diversities of patients aged 30 to 69 years old increased significantly at an average rate of 77% for each age group as the patient age increased. The group aged 70 to 100 years showed the highest diversity level of 55.55 events/arm (95% CI 49.93-61.17, SE 2.867), which is approximately 3.44 times greater than the 20 to 29 years-old young adult group (*P<*.001). Clinical trials that recruited older patients showed significantly higher levels of adverse event diversity, and clinical trials with children younger than 20 years old also have a higher level of adverse event diversity in comparison to younger adults.

**Figure 4:**
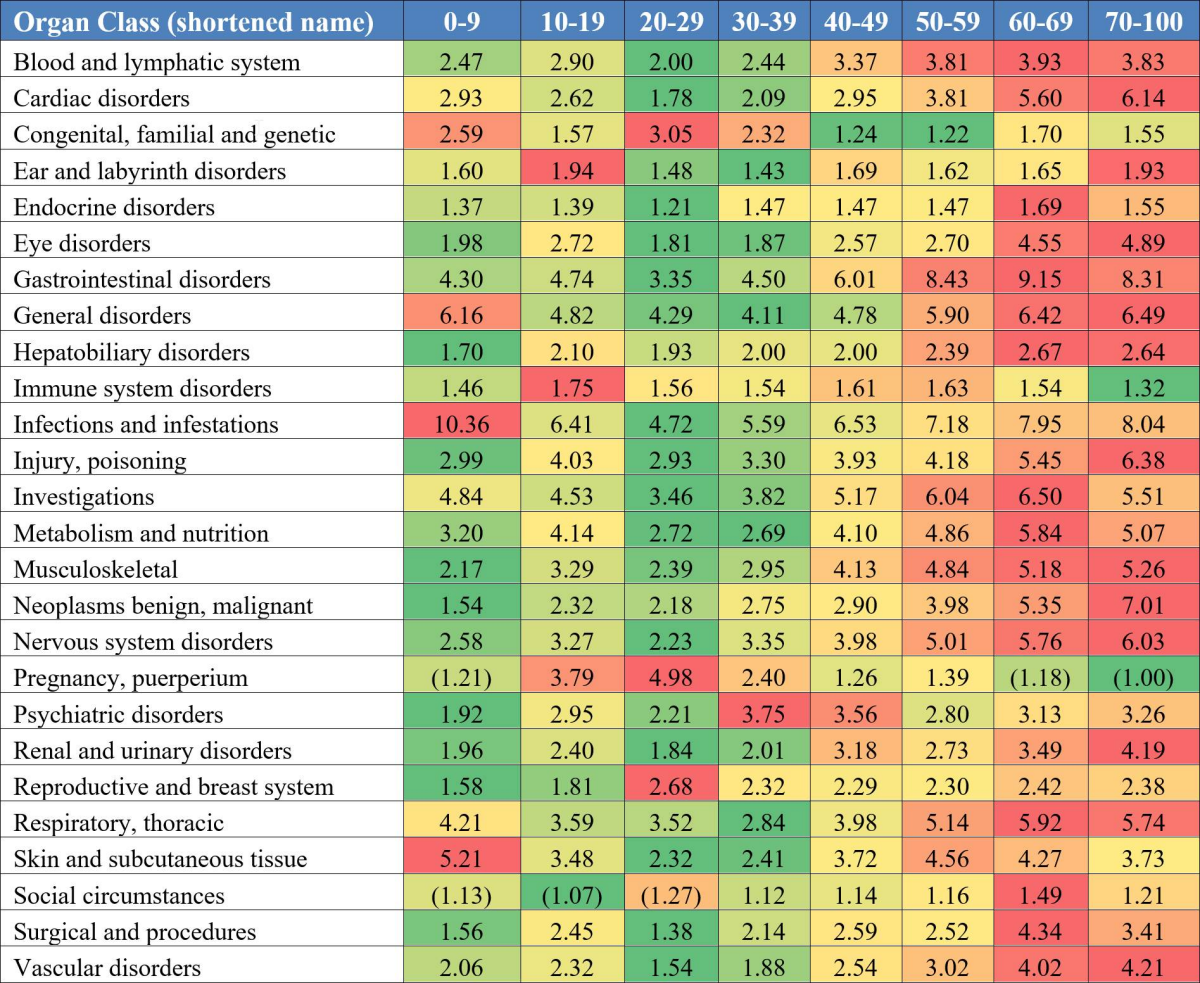
Average adverse event diversity in Medical Dictionary for Regulatory Activities (MedDRA) organ classes across different age groups.

In Figure 4, the adverse events were classified into the 26 MedDRA SOCs. We analyzed the adverse event diversity in each of the age groups and SOCs. Note that event diversity values with low trial supports are shown in brackets; these events were documented in less than 30 clinical trials. Figure 4 displays a heat map of the results of the SOCs diversity analysis. Adverse event diversity is compared across the age groups in different organ categories in the same row. Higher diversity in the same category (ie, on same data row) is shown in red; lower diversity in green. The color intensity is rendered according to the percentile of diversity value when compared to the highest or lowest value. The overall pattern is similar to the previous analysis in which older patient groups (aged 50 years and older) generally showed more types of adverse events. However, when analyzing the diversity level in individual SOCs, we can observe some distinct patterns across the age groups. For example, the 0 to 9 years-old group has a high diversity level of adverse events in infections and infestations (10.36 events/arm), general disorders (6.16 events/arm), and skin and subcutaneous tissue disorders (5.21 events/arm) when compared to young adult group. The adverse events patients in the 10 to 19 years-old group are more likely to experience include ear and labyrinth disorder (1.94 events/arm); immune system disorders (1.75 events/arm); and pregnancy, puerperium, and perinatal conditions (3.79 events/arm) when compared to all other groups. The 20 to 29 years-old group is more diverse in congenital, familial, and genetic disorders (3.05 events/arm) and reproductive system and breast diseases (2.68 events/arm) and higher in pregnancy, puerperium, and perinatal conditions (4.98 events/arm). The 30 to 39 years-old group is more diverse in congenital, familial, and genetic disorders (2.32 events/arm) and pregnancy disorders (2.40 events/arm) and notably higher in psychiatric disorders (3.75 events/arm). The 40 to 49 years-old group also has a high level of event diversity in psychiatric disorders (3.56 events/arm). The three groups of patients older than 50 years generally have a higher event diversity than younger groups, except in the SOCs immune system; congenital, familial, and genetic disorders; and, as expected, pregnancy conditions.

**Figure 5:**
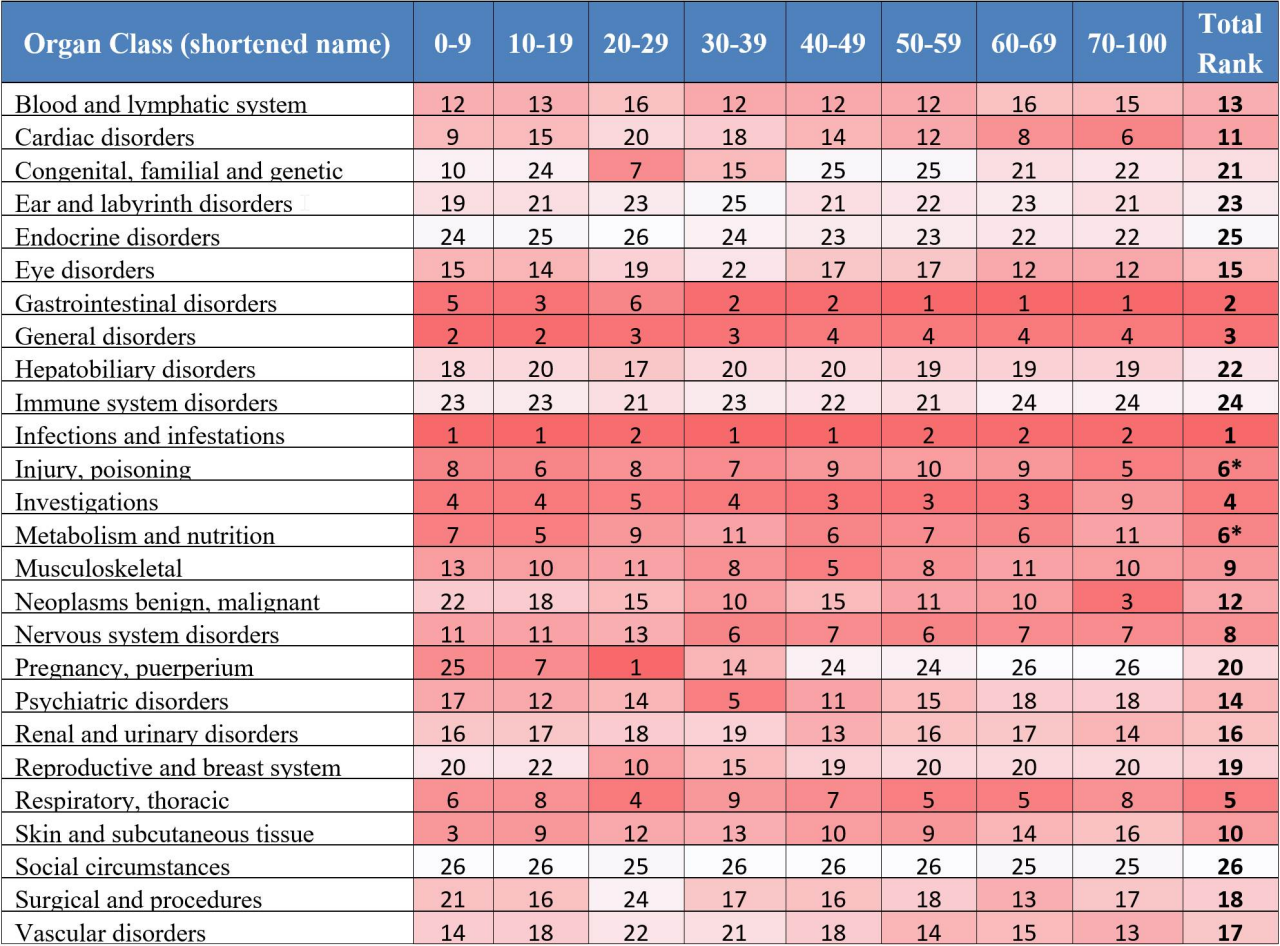
Ranking of average adverse event diversity in each of the age groups. The rankings are calculated within each group. Two organ classes are tied at the sixth place in the total rank (marked with asterisk).

Figure 5 shows the ranking of adverse event diversity in each of the age groups. The ranking is compared in the same age group (ie, same column) and ranked from 1 with highest diversity to 26 with lowest diversity across the 26 categories. The last column on the right shows the total rank of each category across all age groups. In each column, highest diversity value is shown in red and lowest is shown in white. Other values are rendered according their normalized value percentile between the highest and lowest value. The total results in Figure 5 show that the infection and infestations category has the highest average diversity level across most of the age groups, followed by gastrointestinal disorders and general disorders. The lowest categories are immune system disorders, endocrine disorders, and social circumstances.

## Discussion

### Principal Findings

We conducted a population study to analyze the adverse event risk among clinical trial participants. This study differs from patient-level adverse event analysis in that we integrated large amounts of clinical trial data to conduct a population-level analysis looking at adverse event risk patterns across different age groups. We found that young pediatric patients and older patients have a higher level of incidence and diversity of adverse events. The total incidence of adverse events in the youngest age group is higher compared with all other groups. Additionally, the incidence rate of adverse events in this group is significantly higher in the infectious event and general event categories. The older adult groups (aged older than 60 years) showed a comparatively higher incidence of cardiac disorders and vascular disorders. When compared across the 26 SOCs, we observed that the diversity of adverse event patterns differs significantly across the age groups. Older patients show a significantly higher level of adverse event diversity in most of the SOCs, while the younger age groups show higher levels within some SOCs.

### Related Studies

Previous studies have focused on the incidence of adverse events in population levels in various clinical settings. The Canadian Adverse Events Study [18] reported an adverse event rate of 7.5% in 2.8 million hospital admissions. Older patients were more likely to be affected by adverse events. The study also suggests that 9250 to 23,750 deaths from adverse events could have been prevented among the 2.5 million admissions to acute-care hospitals in Canada. A study on 1000 discharged patient records showed that elderly patients (aged 65 years and older) had a high incidence of adverse drug events (18.7%) [19]. Among the identified events, 35% were considered preventable and 32% were serious events. A systematic review of 8 studies [20] on in-hospital adverse events in 6 countries shows that the median incidence of adverse events was 9.2%, and about 43.5% of the adverse events could be preventable. In the outpatient setting, a study showed that adverse event–related visits increased between 1995 and 2005 [21]. Furthermore, the incidence of adverse events also increases with patient age. This study indicated that patient age was one of the important risk factors for adverse event–related visits. Patients aged 65 years and older had a peak of adverse event visits of 47 per 1000 patients. A pediatric study [22] showed that adverse events occurred in about 1% of the pediatric hospitalizations, of which about 0.6% were preventable events compared with a rate of 1.5% in nonelderly adults. The Critical Care Safety Study [23] showed that among 391 studied patients, 20.2% were affected by 120 adverse events and 54% of the events were preventable.

Compared to these studies which focused on preventable adverse events in health care settings, the adverse event rate in clinical studies is significantly higher in terms of incidence rates in all age groups at an average of 27.0%. Many clinical study interventions are experimental in nature and thus are associated inherently with a higher level of risk than normal clinical interventions. In-hospital treatments normally use matured intervention protocols that use validated postmarketing drugs or procedures, whereas clinical trials are often designed to test experimental interventions. For example, in clinical trials aimed to develop new drugs, only about 1 in 10 will be approved by the US Food and Drug Administration [24,25]. Many trials are canceled in the process or the tested substance is disapproved due to risk of adverse events. This suggests that adverse event risk estimation is critical for clinical study preparation. This study provides a quantitative reference for clinical investigators to estimate the trial adverse event risk for targeted age groups when planning clinical trials.

### Clinical Trial Adverse Events and Participant Age

Age is one of the most commonly used clinical study recruitment criteria [26,27], and the risk for adverse events is a primary criterion for evaluating the safety of the targeted intervention in a clinical study [28]. However, few systematic studies have explored the association between adverse clinical trial outcomes and participant age. This study fills the gap by focusing on the adverse event patterns in clinical trials at the population-health level. This study shows that age-related adverse events could be an important factor for clinical trial planning, recruitment, and monitoring. Furthermore, the importance of recruiting more children in clinical trials has been discussed in various reviews [29,30]. The risk of adverse events in children is higher, as suggested by our study results; however, even though numerous regulations have been established to improve children’s safety in clinical studies, there is still a lack of evidence-based support to help clinical investigators estimate the adverse event risks for children at the early stages of a clinical study [28,31]. This study suggests that the adverse event distribution shows strong categorical patterns among age groups, providing a population baseline for estimating the risk of adverse events. Similarly, many studies have verified that older patients have a higher risk of adverse events. Our study shows that among older populations, not only is the adverse event incidence rate higher, the diversity of adverse events also is significantly higher in clinical trials. Furthermore, specific adverse events may be more common in one age group compared to another as seen with the higher incidence of infectious events in the young children group or the peak of psychiatric disorders in the middle age group.

### Limitations and Future Work

This study is limited due to the data granularity on ClinicalTrials.gov. The report on ClinicalTrials.gov does not include adverse event diversity at the individual patient level; for example, we cannot determine how many different adverse events occurred in an individual patient. Therefore, we performed the adverse event diversity analysis on the trial arm level and categorized events by the MedDRE organ classes. The inability to identify individual patients may also create bias when a patient joins multiple trials, although we estimate the proportion of patients joining multiple trials is low because most trials exclude patients who are participating in other trials concurrently. Furthermore, certain types of studies may be more common in one age group than another which could lead to a higher incidence of a type of adverse event. For instance, perhaps few psychiatric studies are performed in the younger patients in comparison to the older patients. For nonserious events, some trials on ClinicalTrials.gov only reported events that exceeded a frequency of 5% within any arm of the trials. This could lead to potential undercount of nonserious events. We used MetaMap [14] to normalized terminologies, which may not normalize terms 100% correctly to the UMLS concepts. However, a few studies evaluated the performance of MetaMap [32,33] and found that the accuracy of MetaMap was over 90%. The MedDRA system classes were updated in March 2016 to include a new category called product issues. The new system class contains events related to device issues. We currently have no adverse events mapped to this category. We also want to compare the differences of adverse event patterns between the intervention groups and the placebo groups on the population level. However, it requires us to develop new natural language processing methods to systematically identify placebo and intervention arms from the free-text trial arm descriptions. This will be our future work.

### Conclusions

The adverse event incidence rate in clinical trial studies is as high as 27.0% at the population level, which is higher than the reported incident rate in various patient care settings (7%-20%). Clinical trials may include a greater risk in terms of adverse events by their nature. Young children and older patients have higher risks of adverse events in clinical trials. The pattern of adverse event types in different organ categories is different across the age groups. Evidence-based risk analysis should be used to facilitate clinical trial design and planning.

## Appendix

Multimedia Appendix 1 Common adverse events that were filtered out.

## Abbreviations

MedDRA: Medical Dictionary for Regulatory Activities
SOC: system organ classes
UMLS: Unified Medical Language System

## References

[1] (2016). ClinicalTrials.gov.

[2] Lazarou J, Pomeranz B, Corey PN (1998). Incidence of adverse drug reactions in hospitalized patients: a meta-analysis of prospective studies. JAMA.

[3] Kaushal R, Bates D, Landrigan C, McKenna K, Clapp M, Federico F, Goldmann DA (2001). Medication errors and adverse drug events in pediatric inpatients. JAMA.

[4] Moore T, Weiss S, Kaplan S, Blaisdell CJ (2002). Reported adverse drug events in infants and children under 2 years of age. Pediatrics.

[5] Rich M, McSherry F, Williford W, Yusuf S, Digitalis Investigation Group (2001). Effect of age on mortality, hospitalizations and response to digoxin in patients with heart failure: the DIG study. J Am Coll Cardiol.

[6] Huddleston J, Wang Y, Uquillas C, Herndon J, Maloney WJ (2012). Age and obesity are risk factors for adverse events after total hip arthroplasty. Clin Orthop Relat Res.

[7] Roland NJ, Bhalla RK, Earis J (2004). The local side effects of inhaled corticosteroids: current understanding and review of the literature. Chest.

[8] Zarin D, Tse T, Williams R, Califf R, Ide NC (2011). The ClinicalTrials.gov results database: update and key issues. N Engl J Med.

[9] Luo Z, Zhang GQ, Xu R (2013). Mining patterns of adverse events using aggregated clinical trial results. AMIA Jt Summits Transl Sci Proc.

[10] Brown E, Wood L, Woods S (1999). The medical dictionary for regulatory activities (MedDRA). Drug Safety.

[11] Trotti A, Colevas A, Setser A, Basch E (2007). Patient-reported outcomes and the evolution of adverse event reporting in oncology. J Clin Oncol.

[12] Edwards I, Aronson JK (2000). Adverse drug reactions: definitions, diagnosis, and management. Lancet.

[13] (1996). International Classification of Diseases, Ninth Revision, Clinical Modification.

[14] Aronson AR (2001). Effective mapping of biomedical text to the UMLS Metathesaurus: the MetaMap program. Proc AMIA Symp.

[15] Aronson A, Lang FM (2010). An overview of MetaMap: historical perspective and recent advances. J Am Med Inform Assoc.

[16] Vincent C, Neale G, Woloshynowych M (2001). Adverse events in British hospitals: preliminary retrospective record review. BMJ.

[17] Classen D, Pestotnik S, Evans R, Lloyd J, Burke JP (1997). Adverse drug events in hospitalized patients and excess length of stay, extra costs, and attributable mortality. JAMA.

[18] Baker GR, Norton PG, Flintoft V, Blais R, Brown A, Cox J, Etchells E, Ghali WA, Hébert P, Majumdar SR, O'Beirne M, Palacios-Derflingher L, Reid RJ, Sheps S, Tamblyn R (2004). The Canadian Adverse Events Study: the incidence of adverse events among hospital patients in Canada. CMAJ.

[19] Kanaan A, Donovan J, Duchin N, Field T, Tjia J, Cutrona Sarah L, Gagne Shawn J, Garber Lawrence, Preusse Peggy, Harrold Leslie R, Gurwitz Jerry H (2013). Adverse drug events after hospital discharge in older adults: types, severity, and involvement of Beers Criteria Medications. J Am Geriatr Soc.

[20] de Vries EN, Ramrattan M, Smorenburg S, Gouma D, Boermeester MA (2008). The incidence and nature of in-hospital adverse events: a systematic review. Qual Saf Health Care.

[21] Bourgeois FT, Shannon MW, Valim C, Mandl KD (2010). Adverse drug events in the outpatient setting: an 11-year national analysis. Pharmacoepidemiol Drug Saf.

[22] Woods D, Thomas E, Holl J, Altman S, Brennan T (2005). Adverse events and preventable adverse events in children. Pediatrics.

[23] Rothschild J, Landrigan C, Cronin J, Kaushal R, Lockley S, Burdick E, Stone PH, Lilly CM, Katz JT, Czeisler CA, Bates DW (2005). The Critical Care Safety Study: The incidence and nature of adverse events and serious medical errors in intensive care. Crit Care Med.

[24] Hay M, Thomas D, Craighead J, Economides C, Rosenthal J (2014). Clinical development success rates for investigational drugs. Nat Biotechnol.

[25] DiMasi J, Feldman L, Seckler A, Wilson A (2010). Trends in risks associated with new drug development: success rates for investigational drugs. Clin Pharmacol Ther.

[26] Luo Z, Yetisgen-Yildiz M, Weng C (2011). Dynamic categorization of clinical research eligibility criteria by hierarchical clustering. J Biomed Inform.

[27] Luo Z, Johnson S, Weng C (2010). Semi-automatically inducing semantic classes of clinical research eligibility criteria using UMLS and hierarchical clustering. AMIA Annu Symp Proc.

[28] Singh S, Loke YK (2012). Drug safety assessment in clinical trials: methodological challenges and opportunities. Trials.

[29] Caldwell PH, Murphy S, Butow P, Craig JC (2004). Clinical trials in children. Lancet.

[30] Knox C, Burkhart PV (2007). Issues related to children participating in clinical research. J Pediatr Nurs.

[31] Shaddy R, Denne SC, Committee on Pediatric Research (2010). Clinical report: guidelines for the ethical conduct of studies to evaluate drugs in pediatric populations. Pediatrics.

[32] Pratt W, Yetisgen-Yildiz M (2003). A study of biomedical concept identification: MetaMap versus people. AMIA Annu Symp Proc.

[33] Osborne J, Gyawali B, Solorio T (2014). Evaluation of YTEX and MetaMap for clinical concept recognition. arXiv.

